# Surface Ocean Biogeochemistry Regulates the Impact of Anthropogenic Aerosol Fe Deposition on the Cycling of Iron and Iron Isotopes in the North Pacific

**DOI:** 10.1029/2022GL098016

**Published:** 2022-07-02

**Authors:** D. König, T. M. Conway, D. S. Hamilton, A. Tagliabue

**Affiliations:** ^1^ School of Environmental Sciences University of Liverpool Liverpool UK; ^2^ College of Marine Science University of South Florida St Petersburg FL USA; ^3^ Department of Earth and Atmospheric Science Cornell University Ithaca NY USA

**Keywords:** anthropogenic iron, iron isotopes, model, ocean, biogeochemistry

## Abstract

Distinctively‐light isotopic signatures associated with Fe released from anthropogenic activity have been used to trace basin‐scale impacts. However, this approach is complicated by the way Fe cycle processes modulate oceanic dissolved Fe (dFe) signatures (δ^56^Fe_diss_) post deposition. Here we include dust, wildfire, and anthropogenic aerosol Fe deposition in a global ocean biogeochemical model with active Fe isotope cycling, to quantify how anthropogenic Fe impacts surface ocean dFe and δ^56^Fe_diss_. Using the North Pacific as a natural laboratory, the response of dFe, δ^56^Fe_diss_, and primary productivity are spatially and seasonally variable and do not simply follow the footprint of atmospheric deposition. Instead, the effect of anthropogenic Fe is regulated by the biogeochemical regime, specifically the degree of Fe limitation and rates of primary production. Overall, we find that while δ^56^Fe_diss_ does trace anthropogenic input, the response is muted by fractionation during phytoplankton uptake, but amplified by other isotopically‐light Fe sources.

## Key Points

- Anthropogenic Fe deposition has a variable impact on surface ocean biogeochemistry which can differ from the pattern of deposition
- Anthropogenic Fe drives distinct responses in primary production, dFe and δ^56^Fe_diss_, dependent on the surface ocean biogeochemical regime
- δ^56^Fe_diss_ traces anthropogenic Fe impact, but requires careful evaluation of other Fe sources and isotope fractionation during Fe uptake

## 1 Introduction

Aeolian deposition is an important source of nutrients to the open surface ocean (Hamilton et al., 2022) especially in remote areas where other sources are limited. In the case of iron (Fe), an essential micronutrient for phytoplankton growth, this atmospheric supply is of particular importance as surface ocean dissolved Fe (dFe) concentrations can be extremely low. Consequently, much research has been devoted to understanding sources, character, transport and dissolution of Fe‐bearing aerosols, with the main focus historically being on Fe input from desert dust (e.g., Baker et al., 2016). By fertilizing past and present surface ocean ecosystems, such dust‐sourced Fe deposition is thought to induce substantial carbon sequestration and to be partly responsible for reduced atmospheric CO_2_ on glacial and millennial timescales (Martin, 1990; Martínez‐Garcia et al., 2011, 2014). However, wildfires and anthropogenic sources (combustion, biomass burning) also release Fe‐bearing aerosols and while the total Fe supply from these pyrogenic sources is lower than for dust (which contributes ca. 95% to total emissions), Fe solubility can be orders of magnitude higher (Ito et al., 2021). This increased solubility may partly be related to the co‐emission of acidic species, which work to enhance the bioaccessibility of this Fe source for phytoplankton (Li et al., 2017).

Isolating the impact of anthropogenic aerosol Fe (anthro‐Fe) on marine biogeochemical cycles requires the disentangling of different aerosol Fe sources. This is challenging as both dust and pyrogenic Fe emissions exhibit high temporal and spatial variability (Hamilton et al., 2020). Furthermore, aerosols often mix during transport, and the atmospheric processing of insoluble minerals to a “soluble” form (referred to as “dFe” hereafter, as it corresponds to oceanic dFe) adds another degree of complexity (e.g., Meskhidze et al., 2019). Mechanistic atmospheric aerosol Fe modeling (Myriokefalitakis et al., 2018), can help trace the origin of marine dFe deposition, whereas geochemical methods allow a more direct assessment of aerosol Fe impacts in the ocean. A recent approach exploits the variable isotopic Fe signatures (δ^56^Fe) of aerosol Fe from different sources, namely the distinctively‐light δ^56^Fe linked to certain anthropogenic combustions processes (up to −4‰, Kurisu et al., 2019), which is in stark contrast with the crustal δ^56^Fe (ca. +0.1‰) observed in dissolvable desert‐dust Fe (Conway et al., 2019; Waeles at al., 2007). These differences in source “endmember” δ^56^Fe have been used to infer the anthropogenic contribution to marine aerosol Fe (Conway et al., 2019; Kurisu et al., 2021) and surface ocean dFe (Pinedo‐González et al., 2020). However, surface ocean dFe isotopic signatures (δ^56^Fe_diss_) are most likely driven by the complex interplay of external sources and fractionation during internal cycling (König et al., 2021), which, together with atmospheric processing, raise uncertainties for using simple mass balance approaches to constraining natural and anthropogenic dFe deposition.

To investigate the effect of anthro‐Fe on surface ocean dFe and δ^56^Fe_diss_, we coupled a novel aerosol dFe deposition scheme to a δ^56^Fe‐enabled biogeochemical ocean model (König et al., 2021). While our modeling approach is global, we focus on the North Pacific as a natural laboratory, both because it receives substantial anthropogenic and natural aerosol Fe input, and due to its contrasting productivity regimes (Fe‐limited subpolar gyre and nitrogen‐limited subtropical gyre; Longhurst, 2007). We find distinct responses of dFe concentration and δ^56^Fe_diss_ to the anthro‐Fe input flux. However, neither response fully corresponds to the anthro‐Fe deposition pattern, due to the diverse biogeochemical state of the surface ocean across the region.

## 2 Methods

We used a version of the PISCES biogeochemical ocean model (Aumont et al., 2015) with variable particle reactivity (Aumont et al., 2017) and a dynamic ligand pool (Völker & Tagliabue, 2015) which incorporates δ^56^Fe cycling by including two prognostic tracers each (heavy ^56^Fe and light ^54^Fe) for dFe, diatom Fe, nanophytoplankton Fe, small and large particulate Fe pools (König et al., 2021). Isotopic fractionation factors (α) are applied to phytoplankton uptake (α of 0.9995), and complexation by organic ligands (α of 1.0006), so that uptake and scavenging (of free dFe) preferentially remove light dFe, and colloidal pumping (of complexed dFe) heavy dFe.

For the three aerosol dFe sources (desert dust, wildfires, and anthropogenic activity), we applied monthly mean dFe deposition fluxes from a 35‐year simulation (1980–2014) of the CAM6 atmospheric model with MIMI Fe mechanism (Hamilton et al., 2020). In addition to tracing the dFe fraction of each Fe source, MIMI accounts for both proton‐ and organic‐ligand dissolution of Fe during transport. Anthro‐Fe emissions are based on an inventory by Rathod et al. (2020), updated to cover the period 1980–2014, and include metal smelting and shipping among other industrial, residential, and traffic Fe. This anthro‐Fe signal neglects dFe released from anthropogenic acidic processing of dust or wildfire Fe, which is instead included in their respective dFe deposition fluxes (assuming no alteration to δ^56^Fe_diss_ during processing). We also included dFe input beneath the surface ocean layer via subsurface dissolution of desert‐dust particles, as described in Aumont et al. (2015), whereas for wildfire and anthro‐Fe only surface dFe deposition is included.

We prescribed the same source δ^56^Fe endmembers as in König et al. (2021): input of moderately‐light hydrothermal dFe (−0.5‰), light to crustal sedimentary dFe (−1‰ to +0.09‰), neutral riverine dFe (0‰), and crustal dust dFe (+0.09‰). For wildfire dFe, we chose a moderately‐light endmember (−0.5‰), based on the generally light values observed in above‐ground plant tissue (Wu et al., 2019 and references therein). For anthro‐Fe, we applied a light endmember (−1.6‰) based on dFe signatures observed for North Atlantic marine aerosol samples likely of anthropogenic origin (Conway et al., 2019).

To determine the impact of anthro‐Fe both on surface ocean Fe cycling and productivity, experiments were run with (“standard” experiment) and without anthro‐Fe deposition, and compared. We also carried out additional experiments to (a) assess the impact of a very light anthropogenic endmember (−4‰) based on δ^56^Fe observed by Kurisu et al. (2019) for dFe from fine aerosol particles sampled close to a steel plant; (b) evaluate the role of surface ocean processing, with model experiments with either phytoplankton uptake or complexation fractionation turned off (i.e., α set to 1); and, (c) determine the respective contribution of each external dFe source to surface ocean δ^56^Fe_diss_ using experiments where the isotopic effect of each source was artificially muted by setting their endmember to 0‰. For an overview of all experiments and their rationale see Table S1 in Supporting Information S1.

All experiments were run off‐line (i.e., a repeating climatological annual cycle of ocean physics) and with identical sedimentary, river, and hydrothermal dFe input for each year. This allows us to isolate dFe deposition from dust, wildfires, and anthropogenic sources as the only cause of interannual variability. Each experiment was spun up for 200 years (1780–1980) using an average monthly deposition field (1980–2014 mean) for natural sources (dust and wildfires). Following Krishnamurthy et al. (2009), we applied a linearly‐increasing scaling factor for anthro‐Fe input from zero emissions in 1880–1980 values, based on the quasi‐linear increase in black carbon emissions during this period (Bond et al., 2007). From 1980 to 2014 monthly varying deposition of dust, wildfire and anthropogenic dFe was used.

## 3 Results and Discussion

### 3.1 Annual dFe Deposition and Impact of Anthro‐Fe

**Figure 1:**
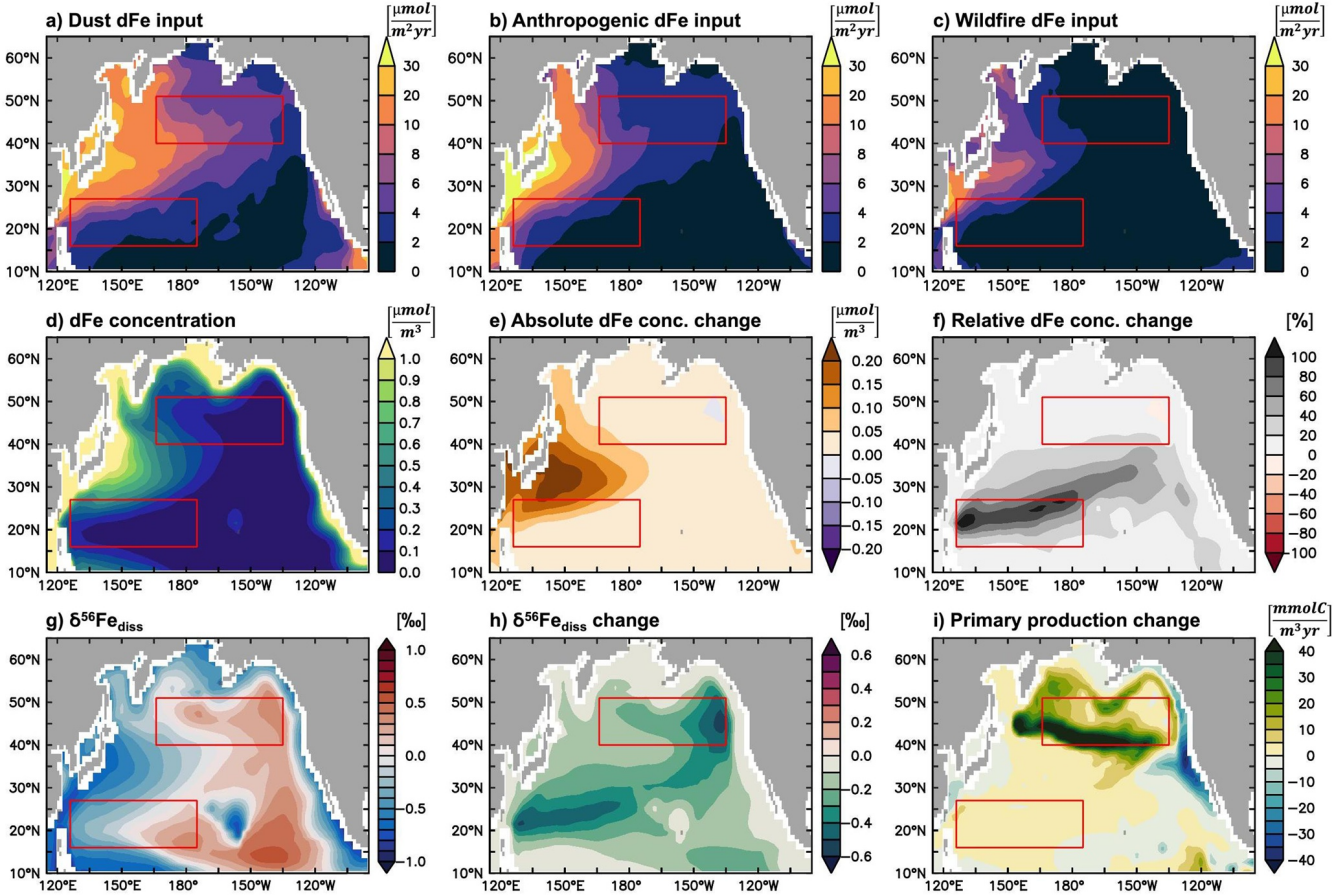
Dust (a), anthropogenic (b), and wildfire (c) dFe deposition fluxes (μmol/m^2^/year) and standard experiment surface ocean (0–10 m) dFe concentration (d; μmol/m^3^) and δ^56^Fe_diss_ (g; ‰) for 2014. Absolute (e; μmol/m^3^) and relative (f; %) change in dFe concentration, in δ^56^Fe_diss_ (h; ‰), and for primary production (i; mmolC/m^3^/year); calculated by subtracting the experiment without anthro‐Fe from the standard experiment (see Table S1 in Supporting Information S1). Red boxes indicate regions analyzed in Section 3.2.

For a representative overview of anthro‐Fe deposition to the North Pacific and the impact on surface ocean biogeochemistry, we focus on model results from 2014. As the last year of our simulations, the accumulated effect on dFe concentrations is highest in 2014, but 2014 signals are very similar to the 2010–2014 average (Figure S1 in Supporting Information S1). As expected, there is a distinct west‐east gradient in aeolian dFe input, dominated by dust (Figures 1a–1c). Nevertheless, the anthropogenic contribution is often around 30% of total aerosol dFe deposition and dominates in the westernmost part of the basin. Wildfire dFe inputs are generally minor in this region (Figure 1c), contributing <20% of total aerosol dFe, but higher in the southwest.

The impact of anthro‐Fe on surface ocean biogeochemistry depends not only on the magnitude of the deposition flux, but also on the local Fe cycle, including the Fe limitation the biota experience. For example, while absolute dFe concentration changes are largest in the west where deposition is highest (Figure 1e), the largest relative change is observed further south and east (Figure 1f), where dFe concentration is typically much lower (Figure 1d), whereas the highest impact on primary production (Figure 1i), is in the Fe‐limited subpolar region.

The impact of anthro‐Fe on δ^56^Fe_diss_ is distinct compared to dFe changes throughout the North Pacific (Figures 1g and 1h). Again, the response of δ^56^Fe_diss_ does not simply follow the atmospheric footprint of anthro‐Fe deposition, but is instead largest in areas with moderate anthro‐Fe input, associated with either a large relative dFe change or a primary production response. While anthro‐Fe addition lowers surface ocean δ^56^Fe_diss_ throughout the region (by up to 0.5‰), δ^56^Fe_diss_ often remains heavy overall, even in areas with a large response to anthro‐Fe (e.g., the eastern subpolar gyre).

### 3.2 Seasonal and Regional Variability

**Figure 2:**
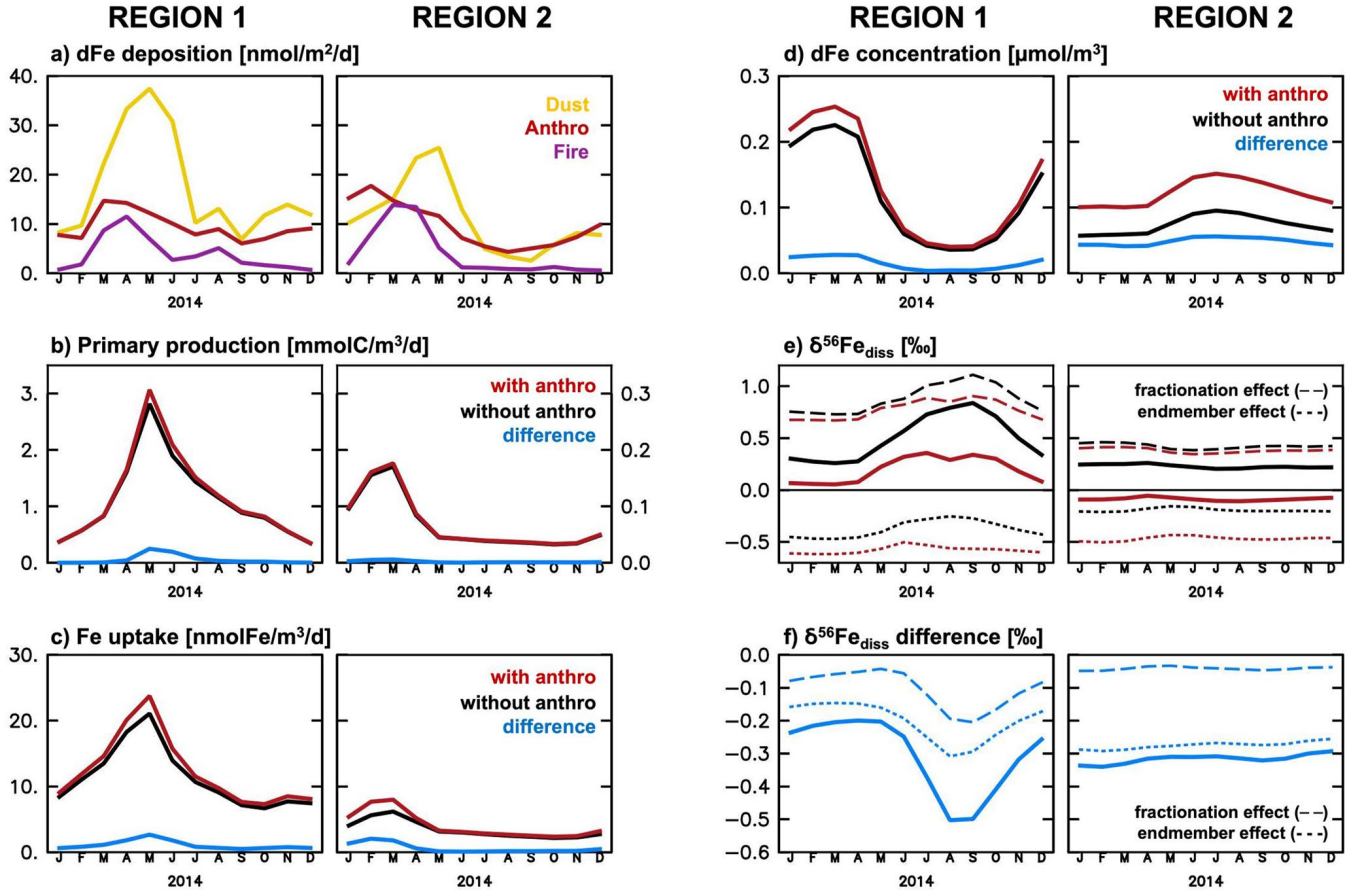
Seasonal variability (year 2014) in dFe deposition (a; nmol/m^2^/day) for a subpolar, Fe‐limited region (Region 1) and a subtropical, nitrogen‐limited region (Region 2; see Figure 1), and the corresponding simulated surface ocean (0–10 m) primary production (b; mmolC/m^3^/day), phytoplankton Fe uptake (c; nmolFe/m^3^/day), dFe concentration (d; μmol/m^3^), and δ^56^Fe_diss_ (e, f; ‰) for experiments with (red) and without (black) anthro‐Fe deposition (difference in blue). For δ^56^Fe_diss_, the contribution of δ^56^Fe fractionation and source endmember effects is shown. See Table S1 in Supporting Information S1 for details on attribution.

To illustrate the seasonal impact of anthro‐Fe deposition we chose two regions with distinct biogeochemistry—one subpolar, Fe‐limited (region 1), and one in the oligotrophic subtropical gyre (region 2; Figures 1 and 2). Despite some regional variability, seasonal trends are broadly consistent within our chosen regions (Figure S2 in Supporting Information S1). In both regions, anthro‐Fe deposition (Figure 2a) amplifies the seasonality in dFe concentration, primary production, and phytoplankton Fe uptake (Figures 2b–2d). This effect is most pronounced for Fe‐limited region 1, which has a strong seasonal cycle in light and mixed layer depth, leading to a replenishment of dFe in winter and a summer drawdown associated with primary production and Fe uptake (Figures 2b–2d; black lines). Here, anthro‐Fe input increases dFe concentration (ca. +4–30 nmol/m^3^; Figure 2c) and Fe uptake rates (ca. +2.7 nmol/m^3^/day, at maximum; Figure 2d) and enhances the early summer primary production peak (Figure 2b; ca. +250 µmolC/m^3^/day), although with little effect or even decreased productivity in late summer. In contrast, there is little response to primary production in oligotrophic (nitrogen‐limited) region 2 (ca. +6 µmolC/m^3^/day, at most; Figure 2b), but, instead, a larger increase in dFe concentration (ca. +50 nmol/m^3^, on average; Figure 2d). In region 2, despite some seasonal changes in Fe uptake, further enhanced by the addition of anthro‐Fe (+2.1 nmol/m^3^/day, at maximum; Figure 2c), the reduced mixed layer cycle causes much smaller seasonality overall than in region 1.

In contrast to the other parameters (Figures 2b–2d), the δ^56^Fe_diss_ seasonality in region 1 is muted by the addition of anthro‐Fe (Figure 2f). In part, this is due to the deposition of isotopically‐light anthro‐Fe in late summer, which decreases the previously very heavy δ^56^Fe_diss_ by over 0.5‰ (Figures 2e and 2f; short dashes). However, 20%–40% of the δ^56^Fe_diss_ decrease is, in fact, due to fractionation effects during internal cycling, as the additional (anthropogenic) dFe supply and the generally weaker productivity in late summer relieves some of the low dFe conditions during which uptake fractionation would otherwise cause very heavy δ^56^Fe_diss_ (Section 3.3). In region 2, the δ^56^Fe_diss_ seasonality remains weak, as anthro‐Fe is responsible for a near constant δ^56^Fe_diss_ decrease of ca. −0.3‰, of which 11%–14% is due to fractionation effects.

Overall, our results indicate that the impact of anthro‐Fe, on both annual and seasonal scales, strongly depends on the underlying biogeochemical state of the upper ocean, as illustrated by the differential responses of the two example regions.

### 3.3 Surface Ocean δ^56^Fe_diss_ Disentangled

**Figure 3:**
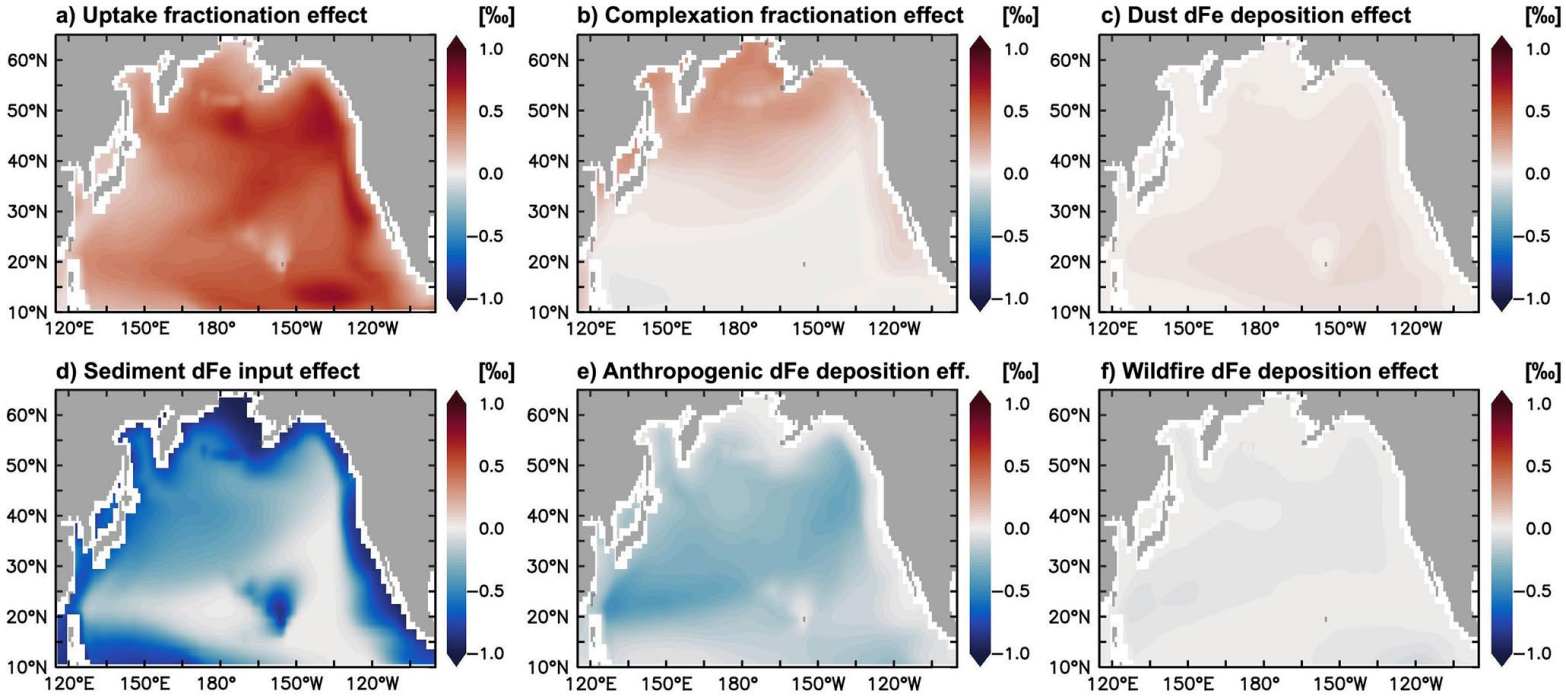
Effect of external dFe sources and fractionating processes on surface ocean (0–10 m) δ^56^Fe_diss_ (a–f; ‰, average for 2014), calculated by subtracting δ^56^Fe_diss_ of experiments with muted δ^56^Fe effects from standard experiment δ^56^Fe_diss_ (see Section 2 and Table S1 Supporting Information S1). Panels (a–f) sum to the overall δ^56^Fe_diss_ distribution (Figure 1g), with a <0.02‰ discrepancy due to hydrothermal dFe.

The dynamics of δ^56^Fe_diss_ in the upper ocean depends on the combination of source δ^56^Fe endmembers and δ^56^Fe fractionation during Fe cycling. In general, the main drivers of the modeled surface ocean δ^56^Fe_diss_ distribution are fractionation during phytoplankton uptake, which drives the δ^56^Fe_diss_ toward heavier values, and dFe input from reducing sediments, which has the opposite effect (Figures 3a and 3d). Complexation fractionation and anthro‐Fe deposition are of intermediate or local importance in our model (Figures 3b and 3e), whereas the impact of dust and wildfire dFe on δ^56^Fe_diss_ are broadly negligible in this region (Figures 3c and 3f).

The strong effect of uptake fractionation on δ^56^Fe_diss_ results from high Fe uptake rates, independent of the degree of Fe limitation, and is largest where dFe concentrations are very low. The large effect of sedimentary dFe on δ^56^Fe_diss_ is due to its pronounced light endmember at shallow depths (−1‰ in the uppermost ca. 400 m; König et al., 2021) and the large dFe input fluxes in coastal areas. Seasonally, the impact of sedimentary dFe is highest in winter as deeper mixed layers entrain additional sediment‐sourced dFe (Figure S3 in Supporting Information S1). Uptake fractionation dominates in late summer, when uptake rates are high and surface dFe has been drawn down to low levels. The effect of complexation fractionation is negligible over most of the region, or even drives δ^56^Fe_diss_ to lighter values in areas where colloidal pumping is the dominant abiotic dFe removal process (König et al., 2021). An exception is the subpolar North Pacific and coastal regions, where elevated scavenging rates, due to high particle and dFe concentrations, mean that complexation is the dominant driver of δ^56^Fe_diss_ (toward heavier values), as only the isotopically‐lighter, uncomplexed dFe is scavenged.

The muted impact of dust dFe on δ^56^Fe_diss_ is due to its (near‐zero) crustal endmember; therefore, dust dFe acts as a “buffer” on δ^56^Fe_diss_. The effect of wildfires on δ^56^Fe_diss_ is also limited compared to other dFe sources due to its low deposition flux in the area and years studied and a relatively‐moderate endmember (−0.5‰), which may be heavier still if soil‐Fe entrainment (with crustal δ^56^Fe) contributes substantially to wildfire Fe (Kurisu & Takahashi, 2019). The impact of the isotopically‐light anthropogenic endmember on δ^56^Fe_diss_ is most pronounced in open ocean areas and in late summer, when the mixed layer is shallowest (Figures 3 and S1 in Supporting Information S1), and increases substantially with a lighter choice of endmember (Section 4.2). This arises from the limited impact of subsurface sedimentary dFe signals in these areas and months. However, even though the impact of anthro‐Fe on δ^56^Fe_diss_ is highest in these summertime open ocean systems, their resultant δ^56^Fe_diss_ is often still heavy (Figure 1g).

Overall, our findings highlight that directly assessing the extent of anthro‐Fe deposition from surface ocean δ^56^Fe_diss_ requires a careful evaluation of other dFe sources and the internal Fe cycling that operate alongside, particularly dFe input from reducing sediments and fractionation during phytoplankton uptake.

## 4 Synthesis and Perspectives

### 4.1 A Mosaic in the Biogeochemical Response to Anthro‐Fe

**Figure 4:**
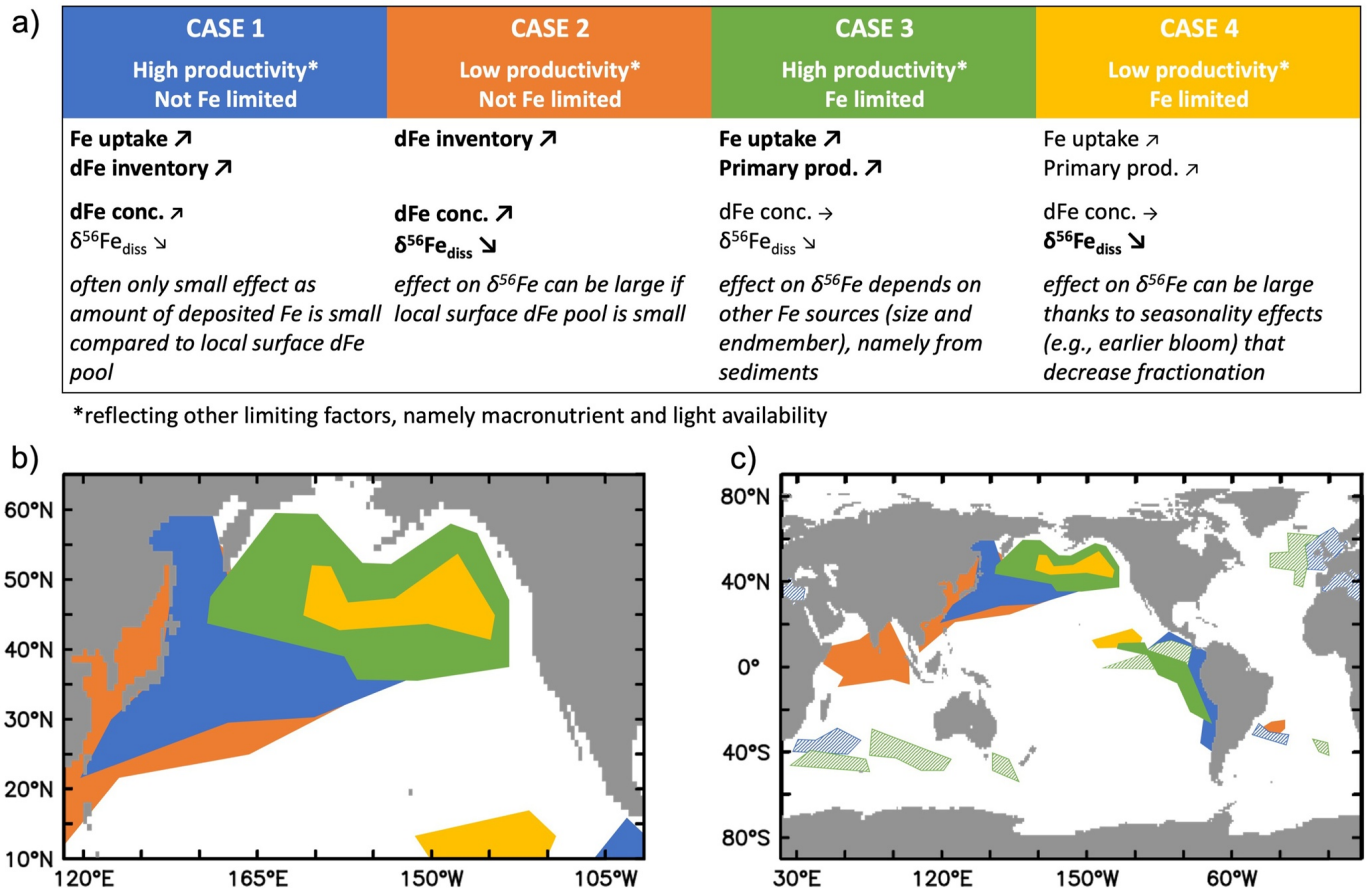
Effect of anthro‐Fe deposition on different ocean biological regimes (a), in the North Pacific (b), and at global scale (c). Note that the depicted regions are illustrative of the present day, and may change if, for instance, nutrient input patterns change (e.g., shifts in aeolian deposition). In hatched areas, the effect of anthro‐Fe is weaker and limited to months of highest productivity.

The model also allows us to assess the varying effect of anthro‐Fe deposition globally (Figure 4). We find that the response of surface ocean systems to anthro‐Fe deposition can broadly be characterized into four different categories based on their underlying biogeochemistry (principally degree of Fe limitation and primary productivity; Figure 4a), as highlighted for the North Pacific (Figure 4b), which receives substantial anthro‐Fe inputs (Figure 1b).

The effects of anthro‐Fe on surface dFe and δ^56^Fe_diss_ varies significantly by category which highlights the importance of the underlying Fe limitation and productivity regime (Figure 4). The mixed layer dFe inventory increases in all non‐Fe limited regions (Cases 1 and 2), compared to Fe‐limited regions (Cases 3 and 4). Conversely, anthro‐Fe deposition stimulates summertime primary production in all Fe‐limited systems (Cases 3 and 4), with a greater impact in Case 3 high‐productivity regimes, but does not stimulate productivity in non Fe‐limited regions (Case 1 and 2). In Case 4 low‐productivity Fe‐limited regimes, the stimulation of productivity by anthro‐Fe is only moderate, and increased productivity in early summer leads to decreased productivity later in the season. In both Case 3 and 4 regimes, dFe concentrations are low and remain unchanged outside unproductive winter months, whereas δ^56^Fe_diss_ decreases, most prominently in Case 4 regimes due to the absence of light sedimentary dFe in these open ocean, low‐productivity areas (Figure 3d) and a dampening of uptake fractionation in late summer when productivity is decreased.

In high‐productivity, non‐Fe limited regions (Case 1), Fe uptake also responds to anthro‐Fe addition, but as this occurs without any stimulation of net primary production it is “luxury” Fe uptake. The extent of luxury uptake depends on how close phytoplankton are to their maximum Fe quota and where luxury uptake is large, it can strongly dampen the increase in dFe inventory so that the dFe concentration increases less than would be expected from anthro‐Fe deposition rates alone. For both Case 1 and 2 non‐Fe limited systems, the tendency of anthro‐Fe to drive lighter δ^56^Fe_diss_ depends both on the magnitude of the dFe pool and the influence of other light dFe sources (notably reducing sediments).

While our focus has been on the North Pacific as a case study, anthro‐Fe deposition, as simulated up to 2014, clearly elicits a response in other regions (Figure 4c). For example, substantial anthro‐Fe deposition to the Northern Indian Ocean leads to an increase in mixed layer dFe inventory, similar to the subtropical North Pacific (Case 2, although Fe limitation may be underestimated by our model in the Arabian Sea; Moffett & Landry, 2020). Primary production and Fe uptake are stimulated in parts of the equatorial Pacific and in the summer months of parts of Atlantic and Southern Indian Ocean, which represent productive, Fe‐limited systems (Case 3), partly due to downstream effects rather than local anthro‐Fe deposition. Downstream effects can also lead to a decrease in productivity as increased nitrogen consumption stimulated by anthro‐Fe can lead to downstream nitrogen limitation, most prominently in the eastern North Pacific. Overall, anthro‐Fe deposition leads to a small increase in global ocean primary production of 0.1% (+0.3% in the uppermost layer), a global increase in Fe uptake of 1.2% (+1.7%), and a dFe concentration increase of 0.3% (+2.4%).

### 4.2 Does Light δ^56^Fe_diss_ Trace Anthro‐Fe Input?

In systems that are not Fe‐limited and show little productivity (due to other limiting factors), light δ^56^Fe_diss_ may be a useful indicator of anthro‐Fe deposition. However, light δ^56^Fe_diss_ can also be related to sedimentary dFe inputs (e.g., Homoky et al., 2009; Severmann et al., 2010), which then need to be thoroughly accounted for when attempting to isolate anthro‐Fe inputs. In higher‐productivity systems, fractionation during Fe uptake drives δ^56^Fe_diss_ toward heavier values in general, so that the “light” δ^56^Fe_diss_ associated with anthro‐Fe input may be masked. Enhanced fractionation in systems where Fe is the limiting nutrient further complicates simple association of δ^56^Fe_diss_ signals with anthro‐Fe deposition, especially as the system responds to the extra dFe supply. Hence, an assessment of productivity and nutrient limitation status is necessary to more fully link surface ocean δ^56^Fe_diss_ signals and anthro‐Fe input. For this purpose, complementary observations may be useful, such as cellular Fe quotas (e.g., Twining et al., 2021), shipboard experiments of Fe uptake (e.g., Boyd et al., 2012) or omics‐based measurements of Fe stress‐induced proteins (e.g., Caputi et al., 2019) or proteomic Fe stress biomarkers (e.g., Saito et al., 2014).

Finally, the assumption of the anthropogenic δ^56^Fe endmember is crucial in estimating anthro‐Fe input based on δ^56^Fe_diss_. Here, we opted for a value (−1.6‰) that, while lighter than any other dFe source in the model, is rather conservative, as some marine‐aerosol δ^56^Fe_diss_ observations are lighter than the combined aeolian‐aerosol δ^56^Fe_diss_ (Figure S4 in Supporting Information S1). Prescribing an even lighter endmember (−4‰) strongly amplifies the effect of anthro‐Fe on surface ocean δ^56^Fe_diss_, rivaling or even exceeding that of sedimentary dFe (Figure S5 in Supporting Information S1), and, for some locations, compares better to the North Pacific surface ocean and aerosol δ^56^Fe_diss_ observations (Figures S4 and S6 in Supporting Information S1). To better constrain the anthro‐Fe endmember, and thus isolate the impact of surface ocean processes, ocean δ^56^Fe_diss_ measurements should be combined with parallel quantification of aerosol δ^56^Fe (e.g., Conway et al., 2019; Kurisu et al., 2021). This would also help in assessing the natural variability of anthropogenic aerosol δ^56^Fe, for instance, between different aerosol size fractions (Kurisu, Sakata, et al., 2016; Kurisu, Takahashi, et al., 2016; Kurisu et al., 2019) and/or effects of atmospheric processing on aerosol δ^56^Fe (Mulholland et al., 2021).

### 4.3 Future Importance of Anthro‐Fe

While the global response to anthro‐Fe deposition in our simulation period (1980–2014) is relatively modest and local, the importance of anthro‐Fe may increase in the future, also depending on changes to other Fe sources. Anthro‐Fe emissions are expected to rise across Asia even if fossil fuel emissions are replaced, as metal smelting already dominates much of the Fe emission source from China and India (Rathod et al., 2020) and is predicted to proliferate as global demand increases. The North Pacific is thus likely to remain a key region in understanding the impact of human activity on ocean biogeochemical cycles. The impact of natural emissions, however, will depend more on climate and human land‐use factors, such as changes in temperature and precipitation which alter aridity, or agricultural expansion which alters vegetation distributions and fire spread. In particular, wildfire activity is generally predicted to increase in extra‐tropical regions (Bowman et al., 2020 and references therein), which provide Fe to the North Pacific, and, as seen for the South Pacific, such changes can have large impacts on ocean biogeochemistry (Tang et al., 2021). Finally, all aeolian dFe deposition is linked with changes to atmospheric circulation and rain patterns (Letelier et al., 2019), as well as air pollution, which affects solubility (Hamilton et al., 2020), whereas entrainment of dFe from other sources, such as sediments, may be linked to changes in ocean circulation and stratification. Thus, the impact of anthro‐Fe is also linked with ongoing natural and anthropogenic climate variability, such as the Pacific Decadal Oscillation in the North Pacific or, on a larger scale, global warming.

## Supporting information

Supporting Information S1

## Data Availability

Model outputs are available from König and Tagliabue (2022) https://doi.org/10.5281/zenodo.5906430.
