# Supplementary material for: Surface Ocean Biogeochemistry Regulates the Impact of Anthropogenic Aerosol Fe Deposition on the Cycling of Iron and Iron Isotopes in the North Pacific
Source: Geophys Res Lett. 2022 Jul 2;49(13):e2022GL098016. doi: 10.1029/2022GL098016 (PMC9539696; doi:10.1029/2022GL098016)
Supplement: Supplementary file 1 — Supporting Information S1 [file GRL-49-e2022GL098016-s001.pdf]

Geophysical Research Letters

Supporting Information for

# Surface Ocean Biogeochemistry Regulates the Impact of Anthropogenic Aerosol Fe Deposition on the Cycling of Iron and Iron Isotopes in the North Pacific

D. König[1], T. M. Conway[2], D. S. Hamilton[3], and A. Tagliabue[1]

[1] School of Environmental Sciences, University of Liverpool, Liverpool, L69 3GP, UK.

[2] College of Marine Science, University of South Florida, St Petersburg, FL 33701, USA.

[3] Department of Earth and Atmospheric Science, Cornell University, Ithaca, NY 14853, USA.

## Contents of this file

Figures S1 to S6
Tables S1

## Introduction

This supporting information includes figures depicting additional information about the model experiments discussed in the main text (Figure S1-6) and a table (Table S1) with an overview of all model experiments, including the rationale behind them.

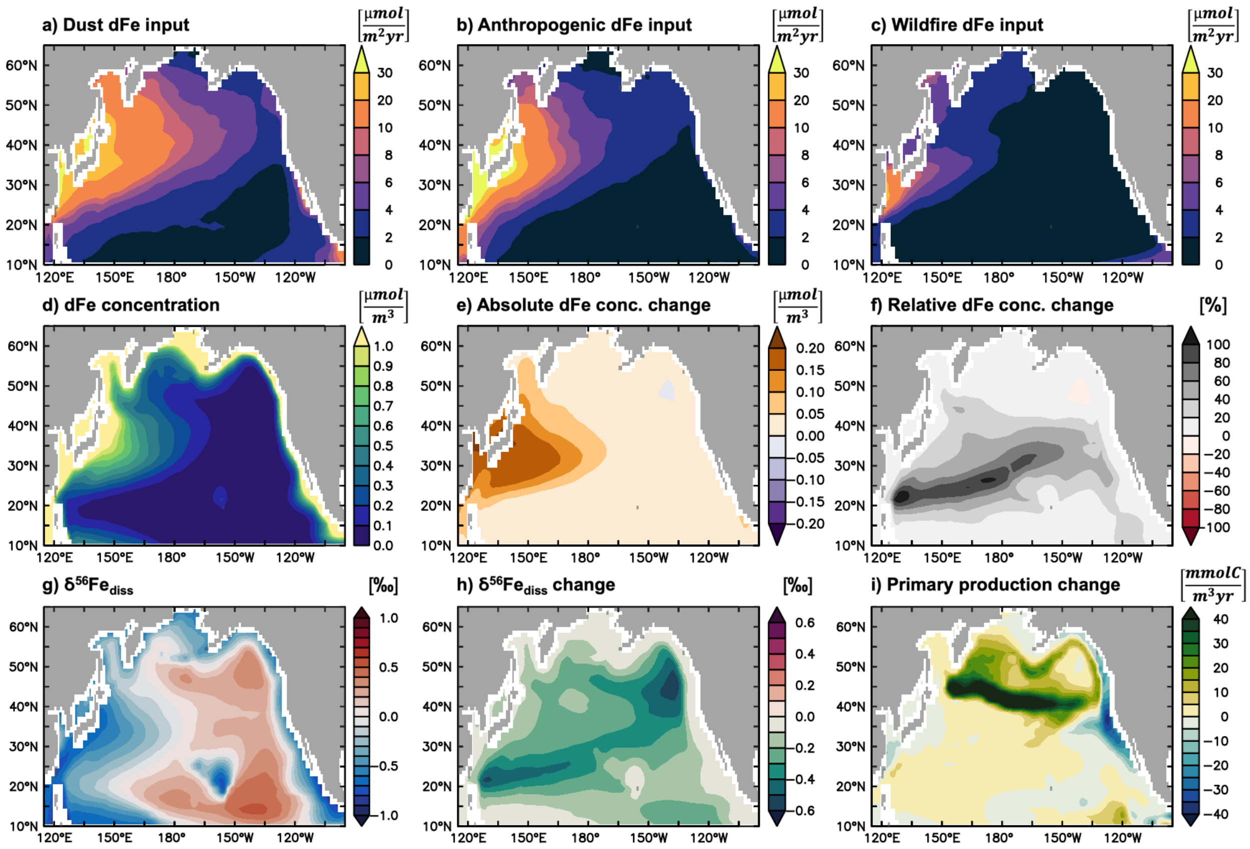


**Figure S1.** 2010 to 2014 average of parameters shown in Figure 1. Note that 2010-2014 is the period with highest anthro-Fe deposition to the North Pacific within the entire study period (1980-2014), and that the 2014 values in Figure 1 are broadly representative for this period.

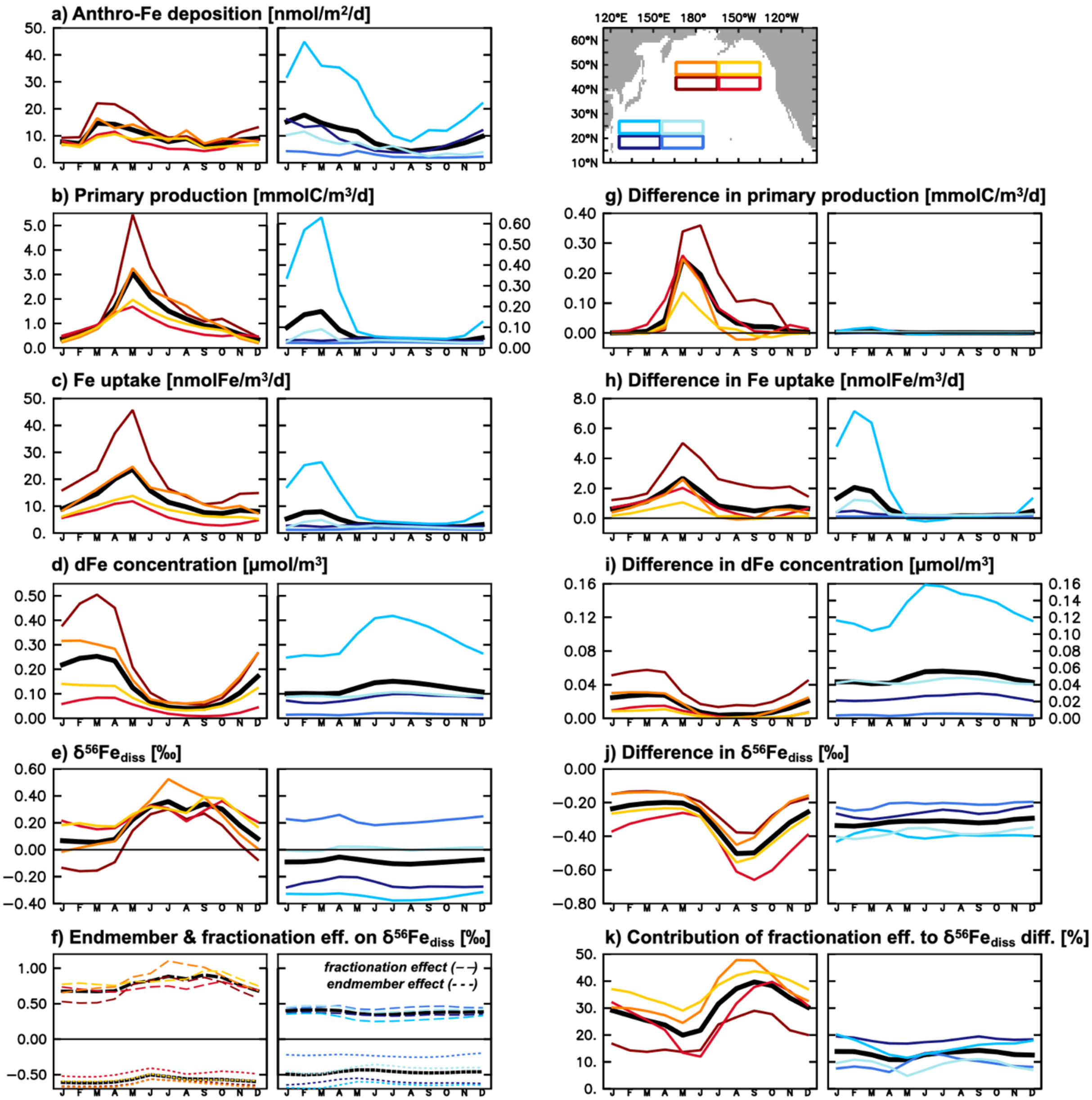


**Figure S2.** Seasonal variability (year 2014) of surface ocean (0-10m) parameters for sub-regions within the subpolar (yellow/red) and subtropical (blue) region depicted in Figure 2. Average of the entire regions are shown in black. Panels a) to f) show absolute values for the standard experiment (i.e., including anthro-Fe deposition). Panels g) to j) show the difference between experiments with (standard) and without (noAnth) anthro-Fe deposition. Panel k) depicts the fraction (%) of the $\delta^{56}Fe_{diss}$ difference (j) that is caused by fractionation effects.

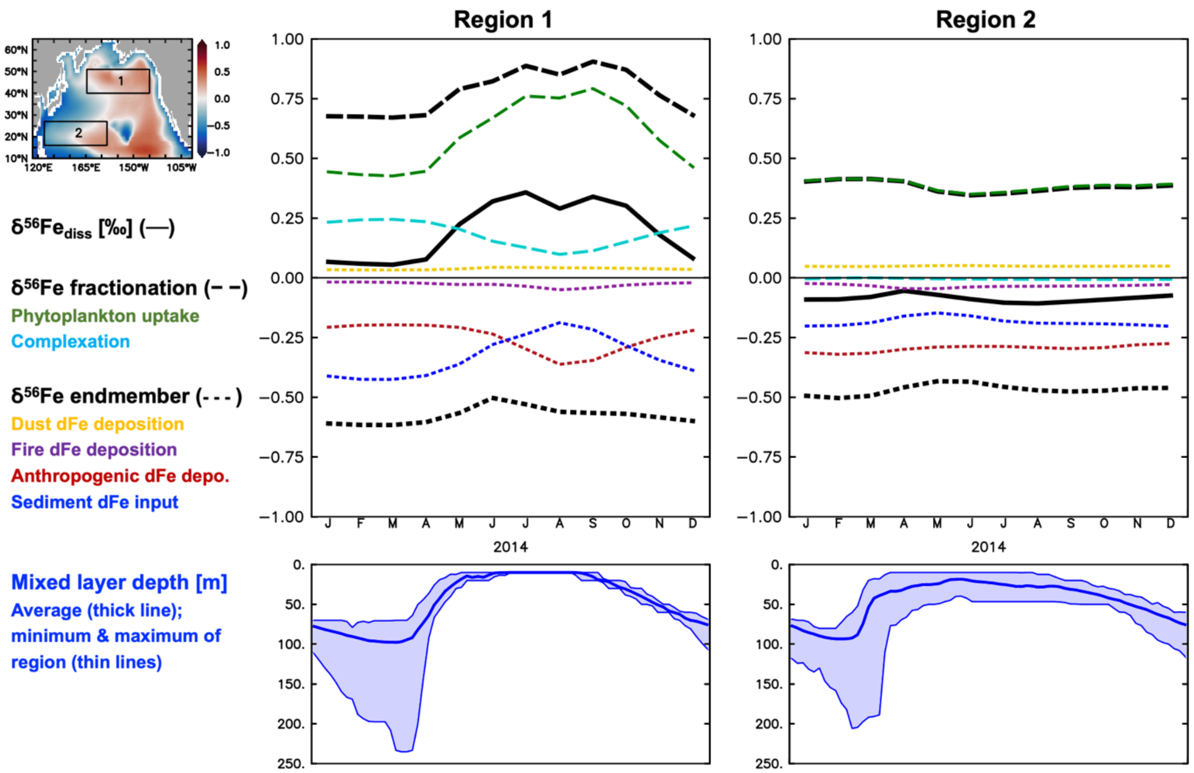


**Figure S3.** Seasonal variability (year 2014) of drivers behind surface ocean $\delta^{56}Fe_{diss}$ and of mixed layer depth, for subpolar Region 1 and subtropical Region 2.

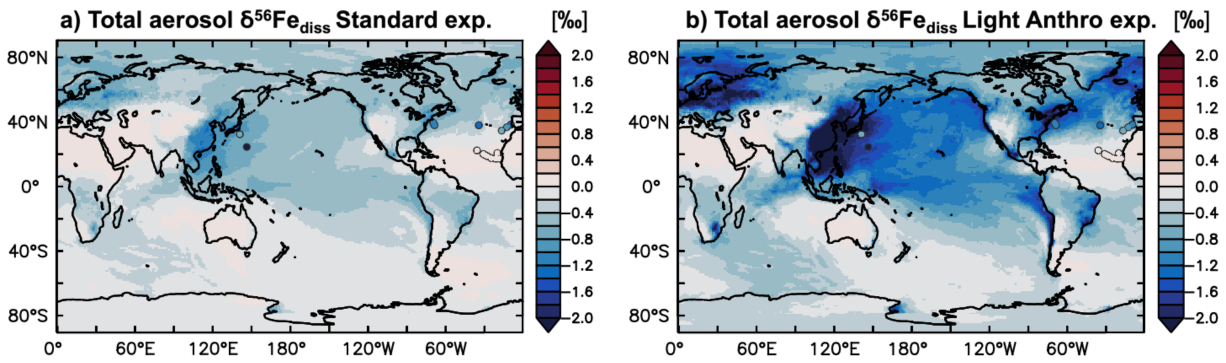


**Figure S4.** $\delta^{56}Fe_{diss}$ of aerosol-dFe (‰; dust, wildfire, and anthro-Fe combined) for the Standard (a; anthro-Fe endmember: -1.6‰) and the Light Anthro experiment (b; anthro-Fe endmember: -4.0‰), as simulated for 2014. Available aerosol $\delta^{56}Fe_{diss}$ observations from Conway, et al. (2019) and Kurisu, et al. (2021) are plotted on top. Note that the light data point of the Pacific dataset (-2.23‰ at around 145°E, 25°N) represents only $\delta^{56}Fe_{diss}$ from fine aerosols (<2.5µm), but is likely representative for bulk aerosol $\delta^{56}Fe_{diss}$, as the fine fraction contained the majority of dFe at this location (Kurisu, et al. 2021).

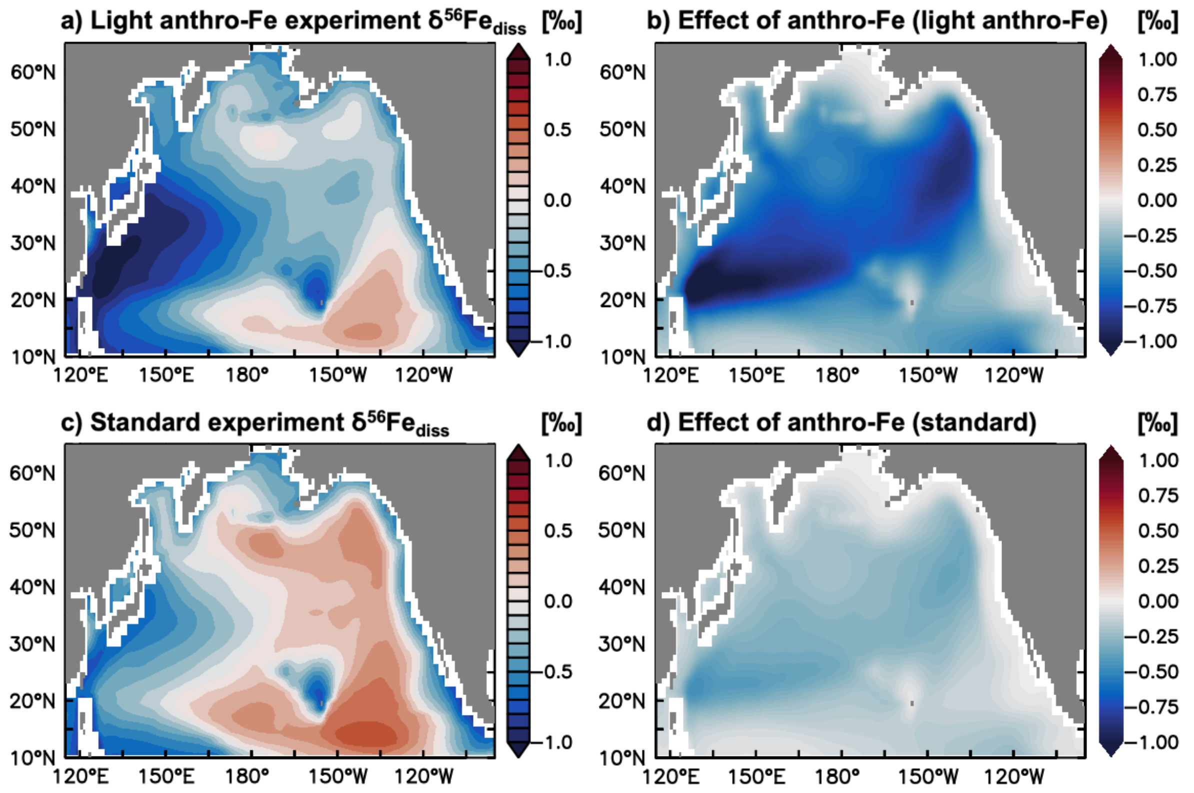


**Figure S5.** Surface ocean (0-10m) $\delta^{56}Fe_{diss}$ (‰) for an experiment with an alternative, lighter anthro-Fe $\delta^{56}Fe_{diss}$ endmember (-4.0‰; a) and the effect of this light endmember on $\delta^{56}Fe_{diss}$ (b; ‰), as simulated for 2014. Panels c and d show the same parameters are for the standard experiment (anthro-Fe endmember: -1.6‰), as a comparison, and are duplicates of Figures 1g and 3e, respectively.

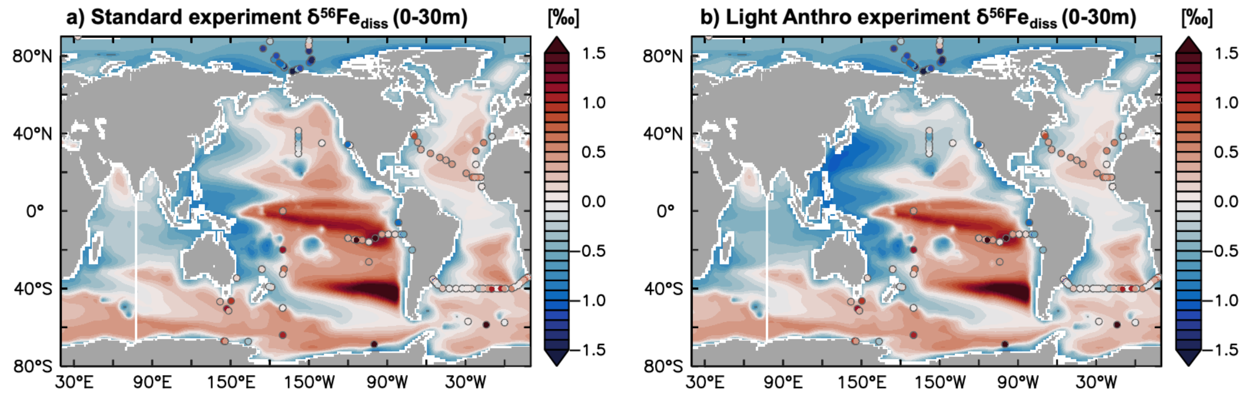


**Figure S6.** Surface ocean (0-30m; to include additional observations) $\delta^{56}Fe_{diss}$ (‰) for the Standard (a; anthro-Fe endmember: -1.6‰) and the Light Anthro experiment (b; anthro-Fe endmember: -4.0‰), as simulated for 2014, with available $\delta^{56}Fe_{diss}$ observations (Abadie et al., 2017; Barrett et al., 2021; Charette et al., 2020; Chever et al., 2015; Conway et al., 2016; Conway & John, 2014, 2015; Ellwood et al., 2015, 2020; Fitzsimmons et al., 2016; GEOTRACES Intermediate Data Product Group (2021); John et al., 2012, 2018; John & Adkins, 2012; Klar et al., 2018; Pinedo-González et al., 2020; Sieber et al., 2021; Summers, 2020; Zhang et al., 2021) plotted on top. Note the choice of anthro-Fe endmember mainly affects the Northern hemisphere where anthro-Fe input is highest (see also Figure S4).

**Table S1.** Overview of model experiments.

| Experiment | Anthro-Fe deposition | $\delta^{56}$Fe source endmembers [‰]* | | | | $\delta^{56}$Fe fractionation fact. | | Rationale |
|---|---|---|---|---|---|---|---|---|
| | | *Anthro.* | *Dust* | *Fire* | *Sediment* | *Uptake* | *Complexation* | |
| **Standard** | Yes | -1.6 | +0.09 | -0.5 | -1 to +0.09 | 0.9995 | 1.0006 | ***Standard experiment set-up*** |
| **noAnth** | No | n.a. | +0.09 | -0.5 | -1 to +0.09 | 0.9995 | 1.0006 | ***Determine overall impact of anthro-Fe deposition (on dFe, $\delta^{56}Fe_{diss}$, primary production, etc.):*** Impact on parameter X = $X_{Standard} – X_{noAnth}$ |
| **neutralAnth** | Yes | 0 | +0.09 | -0.5 | -1 to +0.09 | 0.9995 | 1.0006 | ***Determine effect of $\delta^{56}$Fe source endmembers on $\delta^{56}Fe_{diss}$*** |
| | | | | | | | | Anthropogenic endm. effect = $\delta^{56}Fe_{diss,\ Standard}$ - $\delta^{56}Fe_{diss,\ neutralAnth}$ |
| **neutralDust** | Yes | -1.6 | 0 | -0.5 | -1 to +0.09 | 0.9995 | 1.0006 | Dust endmember effect = $\delta^{56}Fe_{diss,\ Standard}$ - $\delta^{56}Fe_{diss,\ neutralDust}$ |
| **neutralFire** | Yes | -1.6 | +0.09 | 0 | -1 to +0.09 | 0.9995 | 1.0006 | Fire endmember effect = $\delta^{56}Fe_{diss,\ Standard}$ - $\delta^{56}Fe_{diss,\ neutralFire}$ |
| **neutralSed** | Yes | -1.6 | +0.09 | -0.5 | 0 | 0.9995 | 1.0006 | Sediment endmember effect = $\delta^{56}Fe_{diss,\ Standard}$ - $\delta^{56}Fe_{diss,\ neutralSed}$ |
| **noUF** | Yes | -1.6 | +0.09 | -0.5 | -1 to +0.09 | 1 | 1.0006 | ***Determine effect of $\delta^{56}$Fe fractionation on $\delta^{56}Fe_{diss}$*** |
| | | | | | | | | Uptake fractionation effect = $\delta^{56}Fe_{diss,\ Standard}$ - $\delta^{56}Fe_{diss,\ noUF}$ |
| **noCF** | Yes | -1.6 | +0.09 | -0.5 | -1 to +0.09 | 0.9995 | 1 | Complexation fractionation effect = $\delta^{56}Fe_{diss,\ Standard}$ - $\delta^{56}Fe_{diss,\ noCF}$ |
| **noUFnoAnth** | No | n.a. | +0.09 | -0.5 | -1 to +0.09 | 1 | 1.0006 | ***Determine contribution** of uptake (UF) and complexation (CF) fractionation to the net effect of anthro-Fe deposition on $\delta^{56}Fe_{diss}$*** |
| **noCFnoAnth** | No | n.a. | +0.09 | -0.5 | -1 to +0.09 | 0.9995 | 1 | UF contribution = ($\delta^{56}Fe_{diss,\ Standard}$ - $\delta^{56}Fe_{diss,\ noUF}$) – ($\delta^{56}Fe_{diss,\ noAnth}$ - $\delta^{56}Fe_{diss,\ noUFnoAnth}$) |
| | | | | | | | | CF contribution = ($\delta^{56}Fe_{diss,\ Standard}$ - $\delta^{56}Fe_{diss,\ noCF}$) – ($\delta^{56}Fe_{diss,\ noAnth}$ - $\delta^{56}Fe_{diss,\ noCFnoAnth}$) |
| **Light Anthro** | Yes | -4.0 | +0.09 | -0.5 | -1 to +0.09 | 0.9995 | 1.0006 | ***Test effect of alternative, lighter anthropogenic endmember*** |

* Hydrothermal endmember (-0.5‰) and river endmember (0‰) are the same for all experiments

** by comparing (uptake and complexation) fractionation effects for experiments with and without anthro-Fe deposition
